# Predictors of alcohol and substance use among people with post-traumatic stress disorder (PTSD): findings from the NESARC-III study

**DOI:** 10.1007/s00127-023-02472-6

**Published:** 2023-05-03

**Authors:** Adriana Dell’Aquila, David Berle

**Affiliations:** 1 Graduate School of Health, University of Technology Sydney, Sydney, Australia; 2 School of Medicine and Psychology, Australian National University, Canberra, Australia

**Keywords:** Post-traumatic stress disorder (PTSD), Alcohol use disorder (AUD), Substance use disorder (SUD), Trauma exposure, Interpersonal trauma, Self-medication hypothesis

## Abstract

**Purpose:**

The self-medication hypothesis suggests people may develop Alcohol Use Disorder (AUD) or Non-Alcohol Substance Use Disorder (NA-SUD) following PTSD as a maladaptive way of coping with PTSD symptoms. Given that an accumulation of trauma experiences and interpersonal trauma increase the likelihood and severity of PTSD, we sought to determine whether the number and type of traumas additionally predict AUD and NA-SUD following PTSD.

**Methods:**

We analysed data from 36,309 adult participants in the National Epidemiologic Survey on Alcohol and Related Conditions-III (NESARC-III) study (*M* = 45.63 years, SD = 17.53, 56.3% female) who were administered semi-structured diagnostic interviews of trauma exposure and PTSD, AUD and NA-SUD symptoms.

**Results:**

Individuals with PTSD were more likely to have an AUD or NA-SUD than those without PTSD. Endorsement of a greater number of traumas was associated with greater odds of having PTSD, AUD, or NA-SUD. Experience of interpersonal trauma was related to greater odds of having PTSD and subsequent AUD or NA-SUD than not experiencing interpersonal trauma. Multiple experiences of interpersonal trauma compared to one interpersonal trauma exposure also increased the odds of having PTSD followed by AUD or NA-SUD.

**Conclusions:**

Interpersonal trauma and multiple experiences of interpersonal trauma may result in individuals turning to alcohol and substances as a way to alleviate intolerable PTSD symptomology, aligning with the self-medication hypothesis. Our findings highlight the importance of ensuring services and support for interpersonal trauma survivors and for those who have experienced multiple traumas given their increased for unfavourable outcomes.

**Supplementary Information:**

The online version contains supplementary material available at 10.1007/s00127-023-02472-6.

## The association between trauma, post-traumatic stress disorder, alcohol use disorder, and substance use disorder: a secondary analysis of NESARC-III data

Post-traumatic stress disorder (PTSD) can develop following exposure to highly distressing or life-threatening experiences, such as military combat, accidents, assaults, and natural disasters [1]. Around 7–9% of individuals will receive a PTSD diagnosis throughout their lifetime [2, 3]. A myriad of impairments are associated with PTSD [4–6], which often co-occurs with other mental and physical health conditions [7, 8]. The number and type of traumas experienced are among the most reliable risk factors for its development [9–11].

The cumulative effects of multiple trauma exposures, named the dose–response effect [9, 12], is well-documented and posits that exposure to numerous separate traumas will increase the risk of developing PTSD [3, 13–15]. In refugee populations, PTSD occurs in 23% of people who have experienced three or fewer traumas but approaches 100% following exposure to 28 or more traumatic events [14, 15]. The literature has identified a cumulative trauma threshold, such that anyone who experiences enough trauma will subsequently develop lifetime PTSD [15–17]. Studies examining this threshold have provided mixed results; however, it appears to range between 4 and 28 traumatic events [15, 16]. Experiencing multiple traumas does not just increase the likelihood of PTSD but is also associated with PTSD and depression severity, psychological distress, psychosis, decreased coping capacity, and a reduced probability of spontaneous remission from PTSD [13, 18–21].

Differing trauma types appear to moderate the relationship between trauma exposure and the risk of PTSD. Interpersonal trauma, such as assault, torture, and combat, enhance the risk and severity of PTSD compared to non-interpersonal trauma, such as natural disasters and accidents [3, 10, 11, 22–24]. For example, PTSD is twice as likely to develop following a terror attack than a motor vehicle accident [11, 23, 25, 26]. Moreover, multiple experiences of interpersonal trauma are particularly pathological in increasing the risk of dissociative disorders and complex PTSD [27]. The elevated PTSD risk in interpersonal trauma may be explained by greater threat appraisal, where humans are perceived as agents of harm [28–30].

Given that trauma type and dose are associated with a greater likelihood and severity of PTSD, they may also be associated with comorbidities seen in PTSD. Comorbidity in PTSD may be a proxy for greater severity and a sign that individuals have not adjusted to their trauma. Several psychiatric disorders commonly co-occur with PTSD. Almost half (46.4%) of those with PTSD also meet the criteria for Alcohol Use Disorder (AUD) or a Non-Alcohol Substance Use Disorder (NA-SUD) [3, 31–33]. This comorbidity is related to increased suicidality, legal issues, violence, and chronic physical health difficulties, as well as reduced social functioning, in comparison with individuals with either condition alone or with a different comorbid condition [34–38]. Individuals with PTSD and any Substance Use Disorder (SUD) also have a greater number of Axis I and II disorders and a risk of relapse [39]. Compared to PTSD alone, individuals with co-occurring PTSD and substance use also report more severe PTSD symptoms, such as avoidance and hyperarousal [40].

Experiencing more traumas is associated with disordered alcohol and substance use, and a wealth of evidence supports that a higher number of traumas in childhood is associated with a greater lifetime risk of developing AUD [41–45]. Cross-sectional studies on Israeli populations have shown that an accumulation of trauma elevates the probability of developing all types of SUDs, including AUD [45, 46]. Walsh et al. [45] reported that PTSD partially accounted for the association between cumulative trauma and alcohol and nicotine dependence. In contrast, a prospective study of an adolescent sample revealed that PTSD did not mediate the relationship between cumulative trauma exposure and binge drinking [47]. While evidence has supported that multiple traumatic experiences enhance the risk of AUD and SUD, ambiguity exists around whether multiple traumatic experiences lead to PTSD and subsequent AUD or NA-SUD.

The relationship between trauma type and problematic alcohol and substance use also remains unclear. Several prospective studies have found that experiencing interpersonal traumas is associated with an increased risk of alcohol and substance abuse [48, 49]. While some reviews have reported increased alcohol and substance abuse and dependence following interpersonal trauma [50–54], several meta-analytic reviews of longitudinal studies have failed to find this association [55–61]. Therefore, it remains unclear whether there is a relationship between interpersonal trauma exposure and disordered alcohol and substance use. The relationship between interpersonal trauma exposure on PTSD preceding AUD or NA-SUD also appears to be under-researched.

The prevailing and most supported theory for the link between PTSD and AUD or NA-SUD is the self-medication hypothesis, which posits that substances are used to cope with and alleviate intolerable interoceptive experiences and PTSD symptomology [60, 61]. In line with learning theory, substances are negatively reinforcing as they temporarily relieve negative emotions, traumatic memories, and other PTSD symptoms [62, 63]. However, individuals may begin to use substances more frequently and continually, precipitating AUD and NA-SUD onset [60, 64, 65]. Longitudinal evidence shows that in those with comorbid PTSD and AUD or NA-SUD, PTSD typically precedes the other two [64, 66–68]. Specifically, veteran and community sample studies have indicated that SUDs are secondary to PTSD, and PTSD may elevate the likelihood of substance dependence [64, 66, 67]. Experimental studies demonstrate that reminders of traumatic events result in greater substance use in individuals with PTSD than in those without PTSD [69, 70]. Similarly, cues of traumatic events cause alcohol cravings in those with PTSD [71].

It is well-established that alcohol and drug disorders highly co-occur with PTSD [32], and evidence supports that more traumas and traumas of an interpersonal nature are associated with alcohol and other substance use as well as PTSD [11, 13, 45, 49]. However, no previous studies have examined whether AUD and NA-SUDs, following PTSD, are associated with a greater number of traumas or particular types of trauma.

The present study aims to investigate whether number of lifetime trauma exposures and type of trauma are associated with the development of AUD and NA-SUD in people with PTSD. First, it was hypothesised that individuals who experience trauma and develop PTSD are more likely to be diagnosed with AUD or NA-SUD (“AUD/NA-SUD”) than individuals who do not develop PTSD following trauma. Second, we hypothesised that the greater the number of traumas experienced, the greater the likelihood of developing PTSD. Our third hypothesis predicted that the greater the number of traumas experienced, the greater likelihood of developing AUD/NA-SUD. For our fourth hypothesis, we predicted that people who have experienced interpersonal trauma would be more likely to meet the criteria for PTSD and subsequent AUD or PTSD and subsequent NA-SUD compared to those who have not experienced interpersonal trauma. Finally, we hypothesised that people who had experienced multiple interpersonal traumas would be more likely to meet the criteria for PTSD followed by AUD/NA-SUD compared to those who had experienced one interpersonal trauma, consistent with the idea that trauma number and trauma type would have a compounding effect in predicting AUD/NA-SUD following PTSD.

## Methods

### Participants and procedure

The present study utilised data derived from The National Epidemiologic Survey on Alcohol and Related Conditions-III [72]. Data access and ethics approval was obtained (ETH21-6253). The sample included 36,309 civilians aged 18 years or older living in the United States (US). Multistage probability sampling was used to randomly select individuals from the target noninstitutionalised population, including individuals living in college dormitories, group homes or quarters, and dormitories for workers. The sample was adjusted for oversampling and non-response and was weighted to ensure the data reflected the US population. Using a computer-assisted interviewing system, trained interviewers conducted face-to-face interviews between April 2012 and June 2013. Information regarding respondents' demographic characteristics, alcohol and substance use history and mental and physical health difficulties were obtained. Respondents provided informed consent and received a USD$90 incentive for survey participation. A strength of the survey was that participants were asked to estimate the age at which trauma exposures, PTSD and AUD/NA-SUD first occurred, allowing the temporal sequencing of variables to be determined.

### Assessment

The Alcohol Use Disorder and Associated Disabilities Interview Schedule-5 (AUDADIS-5) is a structured diagnostic interview designed for use by non-clinical interviewers. The questions within this interview correspond to the Diagnostic and Statistical Manual of Mental Disorders, Fifth Edition [1] (DSM-5) criteria for a range of psychiatric conditions. It has been demonstrated that each diagnostic category has test–retest reliability within the fair range; for additional psychometric properties, readers are referred elsewhere [72–74].

#### Post-traumatic stress disorder symptoms

A series of binary items corresponded to each DSM-5 symptom of PTSD [1]. A proxy DSM-5 diagnosis was created from the PTSD items, which needed to include associated impairment and/or distress.

#### Trauma number and type

Participants were asked about 19 potentially traumatic events (PTEs) they may have been directly exposed to and 13 PTEs they may have indirectly experienced. Indirect exposure included learning about or witnessing a traumatic event. Endorsement of a stressful life experience, including kidnapping and sexual and physical abuse, was considered to reflect interpersonal trauma exposure ([27]; see Supplementary Information 1 for classification list). Participants that reported experiencing more than four traumatic events were asked to nominate their four most distressing traumas. Hence, the maximum number of traumas recorded for an individual was four, each being of a distinct trauma type.

#### Alcohol and substance use disorder symptoms

The diagnostic questionnaire assessed AUD and NA-SUD according to DSM-5 criteria. Eleven separate substances were evaluated, including cocaine, amphetamines, and heroin. All past-year non-alcohol drug disorders were collapsed to generate one overarching past-year NA-SUD variable. Nicotine, club drugs and solvents or inhalants were not included in the present study for consistency with other published literature [35, 46, 75].

#### Other psychiatric disorders

The AUDADIS-5 was utilised to assess lifetime and past-year DSM-5 mood disorders, such as major depressive disorder (MDD) and anxiety diagnoses, including generalised anxiety disorder, specific phobia, social anxiety disorder, panic disorder and agoraphobia. All past-year anxiety disorders were collapsed into a singular past-year anxiety disorder variable.

#### Demographic variables

Sex, age, race (White, Black, First Nations, Hispanic and Asian, which included Native Hawaiian and Other Pacific Islanders), educational level, and marital status were evaluated.

### Analysis

All analyses were conducted using the Statistical Package for the Social Sciences (SPSS) version 28. The overall significance level for all statistical tests was set at *p* < 0.05 (two-tailed). To test the first hypothesis, a Chi-square Test of Independence was conducted to evaluate associations between current PTSD and past-year AUD and past-year NA-SUD in those who had experienced trauma. For the interested reader, Table 1 is reproduced as a sensitivity analysis in Supplementary Table 1, whereby the age of onset of PTSD was not required to be at least 1 year beyond that of AUD and NA-SUD, respectively, but instead “0” years (on the basis that ages of onset data were rounded to the nearest year) The remaining hypotheses were assessed using Binary Logistic Regressions as a means of examining which variables significantly predicted a diagnosis of PTSD (Hypothesis 2), past-year AUD and NA-SUD (Hypothesis 3), and PTSD followed by AUD/NA-SUD (defined as an earlier age of onset for PTSD than AUD/NA-SUD; Hypothesis 4 and Hypothesis 5). The number of traumas variable was categorised as either 0, 1 or more than one, given that the data set did not allow identification of the precise number of traumas experienced and given the unreliability of participant estimates regarding the number of experienced traumas for repeated and large number of trauma exposures. As at least one trauma experience is required for a diagnosis of PTSD, hypotheses involving the measurement of PTSD compared one to more than one trauma. We controlled for demographic characteristics and past-year MDD and anxiety disorders, consistent with published studies [76, 77].

**Table 1:**
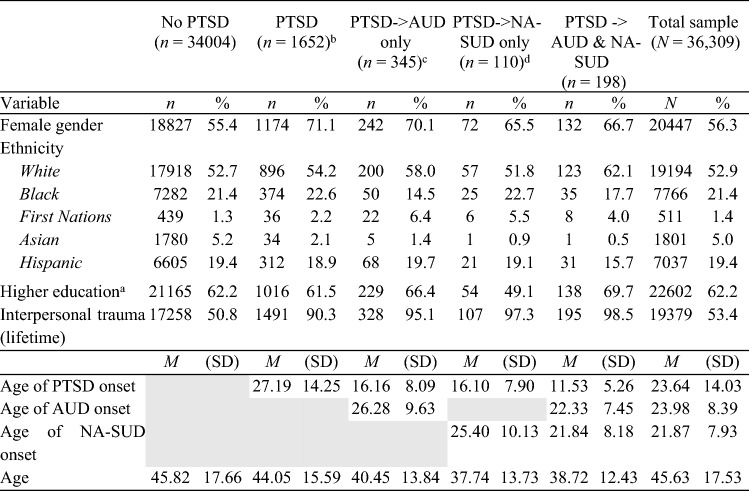
Demographic characteristics and descriptive statistics

*AUD* alcohol use disorder, *PTSD* post-traumatic stress disorder, *NA-SUD* non-alcohol substance use disorder

^a^Completion of a post-school qualification

^b^This group does not include people who developed either AUD or NA-SUD after PTSD

^c^Refers to co-occurring PTSD and AUD, where PTSD preceded the onset of AUD

^d^Refers to co-occurring PTSD and NA-SUD, where PTSD preceded the onset of NA-SUD

## Results

Table 1 summarises the sample demographic characteristics (mean age = 45.63, SD = 17.53, 56.3% female). Interpersonal trauma experiences were reported by 53.4% of the overall sample.

### Post-traumatic stress disorder, alcohol and substance use disorder

A Chi-square test revealed a significant association between past-year PTSD and past-year AUD (*χ*^2^ = 227.72, *p* < 0.001). Specifically, compared to those that experienced trauma and did not develop PTSD, those with PTSD following trauma were more likely to have a past-year AUD (13.32% and 25.19%, respectively). Past-year PTSD was also related to past-year NA-SUD (*χ*^2^ = 525.13, *p* < 0.001): individuals that experienced trauma and had PTSD were more likely to have an NA-SUD than those that experienced trauma and did not develop PTSD (13.38% and 3.28%, respectively).

### Trauma features, post-traumatic stress disorder, alcohol and substance use disorder

Logistic regression analyses were conducted to assess Hypotheses 2–4 (Table 2). The variance inflation (VIF) statistic was reviewed by running a linear regression to examine the multicollinearity assumption. No VIF values were greater than 1.09, indicating no issues with multicollinearity. Linearity was evident for all variables except age.

**Table 2:** Association between multiple experiences of trauma on past year post-traumatic stress disorder, alcohol use disorder and substance use disorder

	*B*	SE	Wald	*df*	*p*	OR	95% CI OR	
							Lower	Upper
*PTSD*								
Number of trauma								
One vs none	0.48	0.05	79.88	1	< 0.001	1.61	1.45	1.79
Age	− 0.01	0.001	66.31	1	< 0.001	0.99	0.99	0.99
Female sex	0.47	0.05	88.81	1	< 0.001	1.60	1.45	1.77
Postschool education	− 0.25	0.05	25.99	1	< 0.001	0.78	0.71	0.86
Marital status (not partnered)	0.19	0.05	15.55	1	< 0.001	1.21	1.10	1.33
Past-year anxiety (met)	1.37	0.05	780.75	1	< 0.001	3.93	3.57	4.33
Past-year MDD (met)	0.84	0.05	244.62	1	< 0.001	2.31	2.08	2.57
Ethnicity (White = reference)								
Black	0.08	0.06	1.84	1	0.175	1.09	0.96	1.22
First nations	0.60	0.14	17.85	1	< 0.001	1.83	1.38	2.42
Asian	− 0.74	0.17	19.54	1	< 0.001	0.48	0.35	0.66
Hispanic	0.13	0.06	4.30	1	0.038	1.14	1.01	1.29
Constant	− 1.08	0.08	183.68	1	< 0.001	0.34		
*AUD*								
Number of trauma								
One vs none	0.56	0.04	218.24	1	< 0.001	1.76	1.63	1.89
More than one	0.59	0.05	122.78	1	< 0.001	1.81	1.63	2.01
Age	− 0.04	0.001	1436.63	1	< 0.001	0.96	0.96	0.96
Female sex	− 0.78	0.03	571.47	1	< 0.001	0.46	0.43	0.49
Postschool education	0.12	0.03	11.26	1	< 0.001	1.12	1.05	1.20
Marital status (not partnered)	0.41	0.03	152.30	1	< 0.001	1.51	1.42	1.62
Past-year anxiety (met)	0.46	0.04	109.86	1	< 0.001	1.58	1.45	1.72
Past-year MDD (met)	0.47	0.05	105.80	1	< 0.001	1.60	1.46	1.75
Ethnicity (White = reference)								
Black	− 0.09	0.04	4.85	1	0.028	0.91	0.84	0.99
First nations	0.31	0.12	6.67	1	0.010	1.36	1.08	1.71
Asian	− 0.44	0.08	28.52	1	< 0.001	0.64	0.55	0.76
Hispanic	− 0.20	0.04	21.52	1	< 0.001	0.82	0.75	0.89
Constant	0.18	0.06	10.42	1	0.001	1.20		
*NA-SUD*								
Number of trauma								
One vs none	0.86	0.08	132.28	1	< 0.001	2.36	2.04	2.74
More than one	0.99	0.10	105.62	1	< 0.001	2.68	2.22	3.24
Age	− 0.04	0.002	407.23	1	< 0.001	0.96	0.96	0.97
Female sex	− 0.79	0.06	192.03	1	< 0.001	0.46	0.41	0.51
Postschool education	− 0.18	0.06	9.51	1	0.002	0.84	0.75	0.94
Marital status (not partnered)	0.57	0.06	85.27	1	< 0.001	1.77	1.57	2.00
Past-year anxiety (met)	0.87	0.07	175.00	1	< 0.001	2.38	2.09	2.71
Past-year MDD (met)	0.79	0.07	134.23	1	< 0.001	2.20	1.92	2.51
Ethnicity (white = reference)								
Black	0.29	0.07	18.98	1	< 0.001	1.34	1.17	1.52
First nations	0.35	0.19	3.18	1	0.074	1.41	0.97	2.06
Asian	− 0.89	0.20	20.92	1	< 0.001	0.41	0.28	0.60
Hispanic	− 0.17	0.08	4.51	1	0.034	0.85	0.73	0.99
Constant	− 1.26	0.10	170.40	1	< 0.001	0.28		

*PTSD* post-traumatic stress disorder, *AUD* alcohol use disorder, *NA-SUD* non-alcohol substance use disorder

#### Trauma number

##### Post-traumatic stress disorder

A logistic regression examined the relationship between the number of traumas and the likelihood of past-year PTSD diagnosis (see the uppermost part of Table 2). The overall model was significant (*χ*^2^ = 1777.23, *p* < 0.001). Experiencing multiple traumas was associated with 1.61 greater odds of having PTSD compared to only having experienced one trauma (*p* < 0.001). Older age and post-school education was related to reduced odds of PTSD (OR = 0.99; OR = 0.78; *p*s < 0.001). Being female, not being partnered and presence of past year anxiety and MDD was associated with increased odds of PTSD (OR = 1.60; OR = 1.21; OR = 3.93; OR = 2.31, respectively; *p*s < 0.001). Compared to people identifying as White, those who were First Nations or Hispanic had greater odds of having PTSD (OR = 1.83; OR = 1.14; *p*s < 0.05); however, Asians had reduced odds (OR = 0.48; *p* < 0.001).

##### Alcohol use disorder

Another logistic regression was used to examine the association between number of traumas and the likelihood of past-year AUD (middle section of Table 2). The logistic regression was significant (*χ*^2^ = 3201.00, *p* < 0.001). Those who experienced one trauma had significantly greater odds of having AUD than those with no reported trauma (OR = 1.76; *p* < 0.001). Similarly, the odds of AUD were greater for those who had experienced two or more traumas in comparison with those without a trauma history (OR = 1.81; *p* < 0.001). Older age and female sex were associated with reduced odds of having AUD (OR = 0.96; OR = 0.46; *p*s < 0.001). Post-school education, not being partnered and past year anxiety and MDD were related to increased odds of having AUD (OR = 1.12; OR = 1.51; OR = 1.58; OR = 1.60; *p*s < 0.001). Compared to people identifying as White, First Nations people (OR = 1.36; *p* = 0.010) had increased odds of having an AUD, while people identifying as Black, Asian, and Hispanic were associated with reduced odds (OR = 0.91; OR = 0.64; OR = 0.82, respectively; *p*s < 0.05).

##### Substance use disorder

The overall regression model could differentiate between those with and without a past-year NA-SUD (*χ*^2^ = 1518.26, *p* < 0.001; see the lower section of Table 2). Individuals exposed to one traumatic event had 2.36 times greater odds of having NA-SUD compared to those with no trauma history (*p* < 0.001). Experiencing multiple traumatic events was associated with 2.68 times greater odds of having an NA-SUD compared to those with no trauma exposure (*p* < 0.001). Older age, female sex and post-school education were related to reduced odds of having an NA-SUD (OR = 0.96; OR = 0.46; OR = 0.84; *p*s < 0.05), whereas not being partnered and past-year anxiety or MDD was associated with greater odds of having an NA-SUD (OR = 1.77; OR = 2.38; OR = 2.20; *p*s < 0.001). Compared to people identifying as White, people identifying as Black (OR = 1.34; *p* < 0.001) had increased odds of having an NA-SUD and the odds decreased if they identified as Asian or Hispanic (OR = 0.41; OR = 0.85; *p*s < 0.05).

#### Number of traumas

##### Post-traumatic stress disorder and alcohol use disorder

A logistic regression was used to ascertain the influence of numbers of trauma on the likelihood of PTSD followed by AUD (upper section of Table 3) was significant (*χ*^2^ = 77.71, *p* < 0.001). Exposure to multiple traumas was not significantly associated with a greater or reduced odds of having PTSD followed by AUD (OR = 0.94), compared to those with no interpersonal trauma exposure (*p* = 0.58).

**Table 3:** Association between multiple trauma exposures and post-traumatic stress disorder and non-alcohol subsequent alcohol use disorder and substance use disorder

	*B*	SE	Wald	*df*	*p*	OR	95% CI OR	
							Lower	Upper
*PTSD before AUD*^a^								
Number of trauma	− 0.06	0.11	0.31	1	0.577	0.94	0.75	1.17
Age	− 0.02	0.00	27.06	1	< 0.001	0.98	0.98	0.99
Female sex	− 0.17	0.11	2.26	1	0.133	0.85	0.68	1.05
Postschool education	0.29	0.11	7.28	1	0.007	1.34	1.08	1.65
Marital status (not partnered)	0.10	0.10	0.95	1	0.330	1.11	0.90	1.36
Past-year anxiety (met)	0.34	0.10	10.73	1	0.001	1.40	1.14	1.71
Past-year MDD (met)	− 0.14	0.11	1.67	1	0.197	0.87	0.70	1.08
Ethnicity (white = reference)								
Black	− 0.47	0.14	11.26	1	0.001	0.63	0.48	0.82
First nations	0.78	0.25	9.70	1	0.002	2.19	1.34	3.58
Asian	− 0.79	0.45	3.05	1	0.081	0.45	0.19	1.10
Hispanic	− 0.15	0.14	1.26	1	0.261	0.86	0.66	1.12
Constant	− 0.49	0.18	7.05	**1**	0.008	0.61		
*PTSD before NA-SUD*^b^								
Number of trauma	− 0.10	0.14	0.45	1	0.502	0.91	0.69	1.20
Age	− 0.02	0.00	29.34	1	< 0.001	0.98	0.97	0.98
Female sex	− 0.31	0.13	5.41	1	0.020	0.73	0.56	0.95
Postschool education	0.00	0.13	0.00	1	0.980	1.00	0.77	1.29
Marital status (not partnered)	0.10	0.13	0.55	1	0.457	1.10	0.85	1.42
Past-year anxiety (met)	0.53	0.13	16.72	1	< 0.001	1.69	1.31	2.18
Past-year MDD (met)	0.00	0.13	0.00	1	0.972	1.00	0.77	1.29
Ethnicity (white = reference)								
Black	− 0.16	0.16	0.93	1	0.334	0.85	0.82	1.18
First nations	0.44	0.31	1.94	1	0.164	1.55	0.84	2.86
Asian	− 10.27	0.74	2.98	1	0.085	0.28	0.07	1.19
Hispanic	− 0.26	0.17	2.30	1	0.129	0.77	0.55	1.08
Constant	− 10.02	0.25	17.21	1	< 0.001	0.36		

*PTSD* post-traumatic stress disorder, *AUD* alcohol use disorder, *NA-SUD* substance use disorder

^a^Refers to co-occurring PTSD and AUD, where PTSD preceded the onset of AUD

^b^Refers to co-occurring PTSD and SUD, where PTSD preceded the onset of SUD

##### Post-traumatic stress disorder and substance use disorder

The predictors reliably distinguished between those with and without PTSD followed by NA-SUD (*χ*^2^ = 83.77, *p* < 0.001; see the lower section of Table 3). The number of traumas was not associated with PTSD followed by NA-SUD (OR = 0.91; *p* = 0.50).

#### Trauma type

##### Post-traumatic stress disorder and alcohol use disorder

The upper part of Table 4 summarises the results of the logistic regression analysis predicting PTSD followed by AUD. Participants who endorsed having experienced an interpersonal trauma had 2.54 times greater odds of having PTSD followed by AUD (*p* < 0.001).

**Table 4:** Association between interpersonal trauma on post-traumatic stress disorder and subsequent alcohol use disorder and substance use disorder

	*B*	SE	Wald	*df*	*p*	OR	95% CI OR	
							Lower	Upper
*PTSD before AUD*^a^								
Interpersonal trauma	0.93	0.25	14.35	1	< 0.001	2.54	1.57	4.11
Age	− 0.02	0.00	25.97	1	< 0.001	0.98	0.98	0.99
Female sex	− 0.19	0.11	2.92	1	0.087	0.83	0.67	1.03
Postschool education	0.25	0.11	5.59	1	0.018	1.29	1.04	1.59
Marital status (not partnered)	0.11	0.10	1.02	1	0.312	1.11	0.91	1.36
Past-year anxiety (met)	0.32	0.10	9.45	1	0.002	1.37	1.12	1.68
Past-year MDD (met)	− 0.14	0.11	1.60	1	0.205	0.87	0.70	1.08
Ethnicity (white = reference)								
Black	− 0.50	0.14	12.78	1	< 0.001	0.61	0.46	0.80
First nations	0.75	0.25	8.85	1	0.003	2.11	1.29	3.46
Asian	− 0.78	0.45	2.96	1	0.086	0.46	0.19	1.12
Hispanic	− 0.16	0.14	1.34	1	0.246	0.85	0.65	1.11
Constant	− 0.89	0.21	17.51	**1**	< 0.001	0.41		
*PTSD before NA-SUD*^b^								
Interpersonal trauma	1.55	0.42	13.48	1	< 0.001	4.72	2.06	10.82
Age	− 0.02	0.00	28.15	1	< 0.001	0.98	0.97	0.98
Female sex	− 0.34	0.13	6.49	1	0.011	0.71	0.55	0.92
Postschool education	− 0.05	0.13	0.15	1	0.701	0.95	0.74	1.23
Marital status (not partnered)	0.11	0.13	0.65	1	0.420	1.11	0.86	1.44
Past-year anxiety (met)	0.50	0.13	15.07	1	< 0.001	1.65	1.28	2.12
Past-year MDD (met)	0.00	0.13	0.00	1	0.979	1.00	0.77	1.31
Ethnicity (white = reference)								
Black	− 0.20	0.16	1.43	1	0.233	0.82	0.59	1.13
First nations	0.40	0.31	1.66	1	0.198	1.50	0.81	2.77
Asian	− 1.27	0.74	2.96	1	0.085	0.28	0.07	1.19
Hispanic	− 0.27	0.17	2.40	1	0.121	0.76	0.54	1.07
Constant	− 1.71	0.32	29.47	1	< 0.001	0.18		

*PTSD* post-traumatic stress disorder, *AUD* alcohol use disorder, *SUD* substance use disorder

^a^Refers to co-occurring PTSD and AUD, where PTSD preceded the onset of AUD

^b^Refers to co-occurring PTSD and SUD, where PTSD preceded the onset of NA-SUD

##### Post-traumatic stress disorder and substance use disorder

Participants who endorsed at least one interpersonal trauma had 4.72 greater odds of having PTSD followed by NA-SUD (*p* < 0.001; lower part of Table 4).

#### Trauma number and type

##### Post-traumatic stress disorder and alcohol use disorder

The overall logistic regression analysis investigating the influence of trauma type and number on the likelihood of PTSD followed by AUD was significant (*χ*^2^ = 102.28, *p* < 0.001; the upper section of Table 5). Experiencing multiple interpersonal traumas increased the odds of having PTSD and subsequent AUD by 1.87-fold compared to experiencing one or less than one interpersonal trauma (*p* < 0.001). Older age was associated with reduced odds of having AUD after PTSD (OR = 0.98; *p* < 0.001). Post-school education and past-year anxiety were related to increased odds of PTSD followed by AUD (OR = 1.29; OR = 1.35, respectively; *p*s < 0.05). Compared to people identifying as White, people identifying as Black (OR = 0.62; *p* < 0.001) had reduced odds of PTSD before AUD, whereas First Nations people had greater odds (OR = 2.05; *p* = 0.004).

**Table 5:** Association between multiple experiences of interpersonal trauma on post-traumatic stress disorder and subsequent alcohol use disorder and substance use disorder

	*B*	SE	Wald	*df*	*p*	OR	95% CI OR	
							Lower	Upper
*PTSD before AUD*^a^								
Multiple interpersonal traumas	0.63	0.13	23.03	1	< 0.001	1.87	1.45	2.42
Age	− 0.02	0.004	23.18	1	< 0.001	0.98	0.98	0.99
Female sex	− 0.20	0.11	3.17	1	0.075	0.82	0.66	1.02
Postschool education	0.25	0.11	5.49	1	0.019	1.29	1.04	1.59
Marital status (not partnered)	0.10	0.11	0.99	1	0.321	1.11	0.90	1.36
Past-year anxiety (met)	0.30	0.10	8.49	1	0.004	1.35	1.10	1.67
Past-year MDD (met)	− 0.15	0.11	1.83	1	0.176	0.86	0.69	1.07
Ethnicity (white = reference)								
Black	− 0.48	0.14	11.73	1	< 0.001	0.62	0.47	0.82
First nations	0.72	0.25	8.16	1	0.004	2.05	1.25	3.37
Asian	− 0.82	0.45	3.30	1	0.070	0.44	0.18	1.07
Hispanic	− 0.14	0.14	1.02	1	0.312	0.87	0.67	1.14
Constant	− 0.71	0.19	14.13	1	< 0.001	0.49		
*PTSD before NA-SUD*^b^								
Multiple interpersonal traumas	0.92	0.18	24.91	1	< 0.001	2.50	1.74	3.58
Age	− 0.02	0.01	25.65	1	< 0.001	0.98	0.97	0.99
Female sex	− 0.36	0.14	7.22	1	0.007	0.70	0.53	0.91
Postschool education	− 0.06	0.13	0.18	1	0.674	0.95	0.73	1.22
Marital status (not partnered)	0.10	0.13	0.58	1	0.446	1.11	0.85	1.43
Past-year anxiety (met)	0.48	0.13	13.74	1	< 0.001	1.62	1.25	2.08
Past-year MDD (met)	− 0.01	0.14	0.01	1	0.944	0.99	0.76	1.29
Ethnicity (white = reference)								
Black	− 0.17	0.17	1.02	1	0.313	0.85	0.61	1.17
First nations	0.36	0.31	1.33	1	0.249	1.44	0.78	2.66
Asian	− 1.32	0.74	3.22	1	0.073	0.27	0.06	1.13
Hispanic	− 0.25	0.18	2.03	10	0.155	0.78	0.55	1.10
Constant	− 1.34	0.25	27.95	1	< 0.001	0.26		

*PTSD* post-traumatic stress disorder, *AUD* alcohol use disorder, *NA-SUD* non-alcohol substance use disorder

^a^Refers to co-occurring PTSD and AUD, where PTSD preceded the onset of AUD

^b^Refers to co-occurring PTSD and NA-SUD, where PTSD preceded the onset of NA-SUD

##### Post-traumatic stress disorder and substance use disorder

The relationship between multiple interpersonal traumas and PTSD before NA-SUD is summarised in the lower section of Table 5. The omnibus model for the analysis was significant (*χ*^2^ = 92.81, *p* < 0.001). Compared to only experiencing one or no interpersonal traumas, those who had experienced multiple interpersonal traumas had 2.50 times greater odds of having PTSD followed by NA-SUD (*p* < 0.001). Older age and female sex were related to reduced odds of having PTSD before NA-SUD (OR = 0.98; OR = 0.70; *p*s < 0.05). Past-year anxiety was related to increased odds of PTSD and subsequent NA-SUD (OR = 1.62; *p* < 0.001).

## Discussion

The current study aimed to affirm the established literature demonstrating the relationship between trauma number and PTSD and resolve the ambiguity surrounding the relationship between trauma number and AUD/NA-SUD. Second, given the absence of literature examining these three factors in tandem, we hoped to determine whether interpersonal trauma and number of traumas were associated with PTSD followed by AUD/NA-SUD. The results strongly suggest that interpersonal trauma is a key factor in predicting PTSD and subsequent AUD/NA-SUD.

In keeping with our first hypothesis, we confirmed that among individuals who have experienced trauma, those with PTSD have higher rates of AUD/NA-SUD than those without PTSD. This finding is consistent with a large body of literature showing that PTSD increases AUD/NA-SUD risk [10, 32, 72, 78]. The results suggest that trauma exposure alone may not be sufficient to cause an individual to develop AUD/NA-SUD. Rather it is the effect of PTSD which may determine whether an individual progresses to problematic alcohol and substance use. These results also align with the self-medication hypothesis, theorising that individuals use alcohol and drugs to alleviate PTSD symptoms.

The study also lends support for the second and third hypotheses: in line with past findings, number of traumas positively correlated with the likelihood of PTSD [9, 14, 15, 20] as well as the likelihood of AUD or NA-SUD [42, 79–81]. Interestingly, number of traumas was not associated with PTSD preceding AUD or NA-AUD, suggesting that while the number of traumas may be relevant for AUD and NA-SUD, number of traumas alone may not predict a transition from PTSD to AUD/NA-SUD. Instead, there may be an independent pathway, whereby an accumulation of traumas may indeed increase the risk of AUD/NA-SUD, but via other, non-PTSD-mediated, pathways. Experiencing multiple traumatic events is associated with more severe mental health conditions such as depression and anxiety and also reduces the ability to cope with and tolerate distress [13, 17, 18, 82, 83]. Therefore, it may be speculated that alternative pathways to AUD/NA-SUD exist, whereby individuals who have experienced many traumatic events develop AUD/NA-SUD after needing to self-medicate symptoms of depression or anxiety.

We also confirmed that interpersonal trauma increased the likelihood of a diagnosis of PTSD and later AUD/NA-SUD compared to those with no reported interpersonal trauma. In addition, our results also supported the exploratory hypothesis that those who have experienced *multiple* interpersonal traumas are more likely to develop AUD/NA-SUD following PTSD compared to individuals exposed to one or less than one interpersonal trauma. Interestingly, once controlling for interpersonal trauma, multiple traumas of any type did not significantly differ from a single trauma experience in predicting PTSD followed by AUD/NA-SUD. This finding suggests that the experience of multiple traumas is most relevant to PTSD and AUD/NA-SUD outcomes when those traumas are interpersonal. These findings are consistent with established literature that has found independent effects of multiple interpersonal traumas on increasing the likelihood and severity of PTSD, AUD, and NA-SUD [22, 25, 84, 85].

Collectively, these results suggest interpersonal trauma exposure is particularly pathological for PTSD and subsequent AUD/NA-SUD development. Interpersonal trauma has been linked to a greater appraisal of threat, subjective distress and pervasive feelings of shame compared to non-interpersonal trauma, which does not typically generate these feelings [23, 29, 30, 86, 87]. Individuals with PTSD are more likely to use substances and alcohol to cope with such negative emotional states than individuals without PTSD [70]. Together, we can postulate that individuals who develop PTSD following interpersonal trauma may experience intense negative emotions, likely exacerbated by multiple interpersonal traumas, hence increasing their inability to cope and the propensity to use alcohol and substances to tolerate severe psychopathology.

While interpersonal trauma and multiple interpersonal traumas were more likely in individuals with PTSD followed by AUD and NA-SUD, the odds ratios suggest this relationship is stronger for NA-SUD than AUD. This is of particular interest as alcohol is more readily and easily accessible, while illicit substances do not have the same ease of access. It could be speculated that individuals with PTSD are desperate to cope with severe PTSD symptomology that follows interpersonal trauma and multiple interpersonal trauma exposure.

As outlined in the results, demographic characteristics and mental health disorders assessed in the study also predicted the likelihood of PTSD, AUD following PTSD, and NA-SUD following PTSD. These included age, sex, post-school education, marital status, ethnicity and past-year anxiety disorder and MDD, confirming findings from previous studies [31, 77, 88–93]. However, the finding that having a post-school education increased the likelihood of AUD was contrary to some studies [94, 95], but consistent with others [96]. Several of these demographic factors controlled for in our models also significantly predicted PTSD followed by AUD/NA-SUD, many of which are yet to be explored in the literature. Future research could elaborate on these further, given the relevance and impacts of co-occurring PTSD and AUD/NA-SUD.

While the current secondary analysis provided critical insights into the association between trauma number and type on PTSD before AUD/NA-SUD, it also has some notable limitations. First, the study utilised a cross-sectional design and was reliant on participant recall regarding the occurrence of events, such as the date of trauma exposure and age of onset of PTSD, AUD and NA-SUD. Furthermore, the determination of PTSD preceding the onset of AUD/NA-SUD was necessarily crude. As age of onset was only reported to the nearest year in the data set, when the age of onset was the same year for both PTSD and AUD/NA-SUD, the participant was not classified as PTSD followed by AUD/NA-SUD. The number of traumas variable also had limitations, whereby participants could only report a maximum of four traumas; second, each trauma had to be a distinct specific trauma type. Thus, repeated experiences of trauma like sexual assault was only counted as one of the four traumas. Nonetheless, assessment of the number of lifetime traumas is notoriously unreliable due to participant recall error and memory distortions [97].

The current investigation has wide-ranging implications. We have shown a temporal sequencing effect, whereby the development of PTSD increases the likelihood of developing an AUD or NA-SUD. These findings impact individuals that, due to their occupation, such as veterans, paramedics and police officers, are likely to be exposed to multiple interpersonal traumas [98, 99]. Specifically, these populations may benefit from access to psychological services to learn adaptive coping strategies to prevent either AUD or NA-SUDs emerging among staff with PTSD, or ideally, the emergence of PTSD in the first place.

To our knowledge, this study is the first to collectively assess the relationship between trauma number and type on PTSD followed by AUD or NA-SUD. The current investigation used a large, generalisable population to identify risk factors predictive of the progression from PTSD to AUD or NA-SUD. These risk factors included experiencing multiple experiences of interpersonal trauma. Our results are broadly consistent with the self-medication hypothesis. Namely, exposure to interpersonal trauma and multiple exposures of an interpersonal nature appeared to be particularly pathogenic for individuals with PTSD, as these risk factors may result in maladaptive attempts to self-medicate, with continued and frequent use precipitating the onset of AUD or NA-SUD. Future studies should aim to replicate these findings using prospective study designs.

## Supplementary Information

Below is the link to the electronic supplementary material.

Supplementary file1 (DOCX 20 KB)

## Data Availability

Researchers can apply for access to data from the NESARC-III study through the National Institute on Alcohol Abuse and Alcoholism (https://www.niaaa.nih.gov/research/nesarc-iii).

## Code availability

Requests for analysis scripts and code can be sent to the corresponding author at david.berle@uts.edu.au.
