# Supplementary material for: Predictors of alcohol and substance use among people with post-traumatic stress disorder (PTSD): findings from the NESARC-III study
Source: Soc Psychiatry Psychiatr Epidemiol. 2023 May 3;58(10):1509–22. doi: 10.1007/s00127-023-02472-6 (PMC10460312; doi:10.1007/s00127-023-02472-6)
Supplement: Supplementary file 1 — Supplementary file1 (DOCX 20 KB) [file 127_2023_2472_MOESM1_ESM.docx]

**Supplementary Table 1**

*Sensitivity check of demographic characteristics when PTSD preceding AUD and SUD is zero years.*

|  | No PTSD  (*n* = 34004) | | PTSD  (*n* = 1556)^†^ | | PTSD->AUD only  (*n* = 394)^††^ | | PTSD->SUD only  (*n* = 117)^‡^ | | PTSD -> AUD & SUD  (*n* = 228) | | Total sample  (*N* = 36,309) | | |
| --- | --- | --- | --- | --- | --- | --- | --- | --- | --- | --- | --- | --- | --- |
| Variable | *n* | % | *n* | % | *n* | % | *n* | % | *n* | % | *N* | % |  |
| Female gender | 18827 | 55.4 | 1115 | 71.7 | 272 | 69.0 | 78 | 66.7 | 155 | 65.1 | 20447 | 56.3 |  |
| Ethnicity |  |  |  |  |  |  |  |  |  |  |  |  |  |
| *White* | 17918 | 52.7 | 838 | 53.9 | 234 | 59.4 | 61 | 52.1 | 143 | 60.1 | 19194 | 52.9 |  |
| *Black* | 7282 | 21.4 | 354 | 22.8 | 63 | 16.0 | 25 | 21.4 | 42 | 17.6 | 7766 | 21.4 |  |
| *First Nations* | 439 | 1.3 | 34 | 2.2 | 22 | 5.6 | 7 | 6.0 | 9 | 3.8 | 511 | 1.4 |  |
| *Asian* | 1780 | 5.2 | 32 | 2.1 | 4 | 1.0 | 2 | 1.7 | 3 | 1.3 | 1801 | 5.0 |  |
| *Hispanic* | 6605 | 19.4 | 298 | 19.2 | 71 | 18.0 | 22 | 18.8 | 41 | 17.2 | 7037 | 19.4 |  |
| Higher education^#^ | 21165 | 62.2 | 946 | 60.8 | 272 | 69.0 | 59 | 50.4 | 160 | 67.2 | 22602 | 62.2 |  |
| Interpersonal trauma (lifetime) | 17258 | 50.8 | 1400 | 90.0 | 373 | 94.7 | 113 | 96.6 | 235 | 98.7 | 19379 | 53.4 |  |
|  | *M* | (SD) | *M* | (SD) | *M* | (SD) | *M* | (SD) | *M* | (SD) | *M* | (SD) |  |
| Age of PTSD onset |  |  | 27.21 | 14.43 | 18.43 | 10.19 | 16.56 | 8.15 | 12.63 | 5.80 | 23.64 | 14.03 |  |
| Age of AUD onset |  |  |  |  | 26.72 | 9.86 |  |  | 22.57 | 8.07 | 23.98 | 8.39 |  |
| Age of SUD onset |  |  |  |  |  |  | 25.14 | 10.10 | 21.29 | 7.90 | 21.87 | 7.93 |  |
| Age | 45.82 | 17.66 | 44.28 | 15.68 | 40.93 | 13.83 | 37.46 | 13.80 | 38.43 | 12.46 | 45.63 | 17.53 |  |

^#^ Completion of a post-school qualification.

† This group does not include people who developed either AUD or SUD after PTSD.

†† Refers to co-occurring PTSD and AUD where PTSD preceded the onset of AUD by as little as zero years.

‡ Refers to co-occurring PTSD and SUD where PTSD preceded the onset of SUD by as little as zero years.

AUD = Alcohol Use Disorder; PTSD = Posttraumatic Stress Disorder; SUD = Substance Use Disorder.
